# The Role of Beta-Hydroxybutyrate in Mitigating the Inflammatory and Metabolic Consequences of Uric Acid

**DOI:** 10.3390/metabo14120679

**Published:** 2024-12-04

**Authors:** Nicole P. Remund, John G. Larsen, Marley J. Shin, Cali E. Warren, Isabelle L. Palmer, Iris J. Kim, Elijah T. Cooper-Leavitt, Derek M. Clarke, Colson G. Beus, Richard J. Johnson, Juan A. Arroyo, Paul R. Reynolds, Benjamin T. Bikman

**Affiliations:** 1 Department of Cell Biology and Physiology, Brigham Young University, Provo, UT 84602, USA; 2 Department of Medicine, University of Colorado, Anschutz Medical Campus, Aurora, CO 80045, USA

**Keywords:** uric acid, inflammation, ketones, insulin resistance, mitochondria

## Abstract

**Background:** Uric acid (UA), a metabolite of purine and fructose metabolism, is linked to inflammation and metabolic disorders, including gout and cardiovascular disease. Its pro-inflammatory effects are largely driven by the activation of the nucleotide-binding oligomerization domain-like receptor family pyrin domain-containing 3 (NLRP3) inflammasome, leading to increased cytokine production. Beta-hydroxybutyrate (BHB), a ketone produced during fasting or carbohydrate restriction, has been shown to reduce inflammation. This study explores the role of BHB in mitigating the inflammatory and metabolic effects of elevated uric acid levels. **Methods:** We utilized a murine muscle cell culture treated with UA and BHB. **Results:** Muscle cells treated with UA had increased production of pro-inflammatory cytokines and reduced cell viability. Co-treatment with BHB reversed these effects, improving cell survival and reducing cytokine levels. Additionally, uric acid impaired mitochondrial function and increased oxidative stress, which were mitigated by BHB. Furthermore, uric acid disrupted insulin signaling, but BHB co-treatment restored insulin sensitivity. **Conclusions:** These findings suggest that BHB holds therapeutic potential by counteracting the inflammatory and metabolic disruptions caused by elevated uric acid, making it a promising target for conditions such as hyperuricemia and metabolic syndrome.

## 1. Introduction

Uric acid is a byproduct of purine and fructose metabolism associated with metabolic disturbances and inflammatory conditions, particularly in the context of hyperuricemia. When elevated, uric acid has been implicated in a range of metabolic disorders, including gout, cardiovascular disease, insulin resistance, and metabolic syndrome [1]. Uric acid has pro-inflammatory effects primarily mediated through the production and release of myriad pro-inflammatory cytokines [2].

Recent research has highlighted the potential role of ketones, particularly beta-hydroxybutyrate (BHB), in mitigating the inflammatory and metabolic effects associated with elevated uric acid levels. Ketones, produced during states of carbohydrate restriction and fasting, have been shown to exert anti-inflammatory effects by inhibiting the nucleotide-binding oligomerization domain-like receptor family pyrin domain-containing 3 (NLRP3) inflammasome [3]. Whether this inhibition is sufficient to prevent the cascade of inflammatory responses typically triggered by uric acid is unknown.

Beyond its well-established role in promoting an inflammatory environment, uric acid is increasingly recognized for its role in disrupting mitochondrial physiology and promoting oxidative stress. Elevated uric acid levels impair the efficiency of ATP production and thus contribute to oxidative stress by generating reactive oxygen species (ROS) [4]. This relationship is particularly pronounced in metabolic syndrome and insulin resistance, where mitochondrial health is already compromised.

Despite evidence that a ketogenic diet may contribute to a temporary rise in uric acid levels [5], these same diets paradoxically result in a reduction in inflammatory markers [6] and robust improvements in mitochondrial efficiency [7]. This suggests there is some variable introduced into the metabolic milieu that opposes the inflammatory and mitochondrial changes induced by uric acid. Indeed, the anti-inflammatory properties of ketones, such as BHB, may outweigh the potential pro-inflammatory effects of mildly elevated uric acid levels. The precise mechanisms behind this paradox remain an area of active investigation, with current hypotheses focusing on the modulation of oxidative stress and the preservation of mitochondrial function by ketones. Moreover, BHB not only serves as an alternative energy source to glucose and other fuels but also modulates key signaling pathways that influence inflammation and mitochondrial bioenergetics [3,7,8]. Studies have demonstrated that BHB can preserve mitochondrial function and reduce reactive oxygen species (ROS) production, thereby potentially counteracting the detrimental effects of uric acid on cellular bioenergetics [7].

This project aims to explore the dual role of ketones in mitigating both the inflammatory and metabolic complications associated with elevated uric acid levels. By examining the molecular mechanisms underlying these interactions, we seek to highlight the therapeutic potential of ketone bodies in conditions characterized by hyperuricemia and associated metabolic disturbances.

## 2. Experimental Design

### 2.1. Cell Culture

C2C12 murine muscle cells were maintained in DMEM (Dulbecco’s modified Eagle’s medium; D6546, Sigma-Aldrich, Saint Louis, MO, USA) plus 10% FBS (Invitrogen, Carlsbad, CA, USA). For β-hydroxybutyrate treatments (β-HB), cells were incubated with 5 mM of β-HB (54965, Sigma-Aldrich) for 24 h. Uric acid was used at 500 µM (U2625, Sigma-Aldrich) over the same time. For insulin stimulation, cells were incubated with insulin (100 nM; Humulin; Eli Lilly, Indianapolis, IN, USA) for 10 min prior to collection.

### 2.2. Cell Viability

Cells were plated at a concentration of 2 × 10^5^ cells/mL in 96-well plates (at 100 μL/well), and cultured for 48 h total with vehicle (water; CON) and uric acid (500 µM), BHB (5 mM), or both (U + B). Cellular viability was determined by MTT assay (Sigma-Aldrich) and measured on a BioTek Synergy 2 plate reader.

### 2.3. NLRP3 Activity

Following treatment, NLRP3 inflammasome activity was measured using the Caspase-Glo 1 Inflammasome Assay (Promega, Madison, WI, USA) according to the manufacturer’s instructions. Briefly, cell supernatants were collected and combined with the assay reagent in 96-well white plates. Luminescence was measured using a microplate reader, with increased luminescence indicating elevated caspase-1 activity and NLRP3 inflammasome activation. Data were normalized to untreated controls and expressed as fold change in activity.

### 2.4. Mitochondrial Respiration

Cells were prepared for mitochondrial respiration as described previously [9,10] using the Oroboros O2K oxygraph (Oroboros, Innsbruck, Austria). Electron flow through complex I was supported by glutamate + malate (10 mM and 2 mM, respectively) to determine leak oxygen consumption (GM_L_). Following stabilization, adenosine diphosphate (ADP) (2.5 mM) was added to determine oxidative phosphorylation capacity (GM_D_). Succinate was added (GMS_D_) for complex I + II electron flow into the Q-junction. To determine full electron transport system capacity in cells over oxidative phosphorylation, the chemical uncoupler carbonyl cyanide 4-(trifluoromethoxy) phenylhydrazone (FCCP) was added (0.05 μM, followed by 0.025 μM steps until maximal O_2_ flux was reached). Lastly, residual oxygen consumption was measured by adding antimycin A (2.5 μM) to block complex III action, effectively stopping any electron flow, which provides a baseline rate of respiration. Following respiration protocol, samples were removed from the chambers and measured for protein content.

### 2.5. H_2_O_2_ Emission

H_2_O_2_ emission was measured using an Amplex Red Hydrogen Peroxide/Peroxidase Assay kit (Invitrogen, A22188) as described previously [11]. In short, 80,000 cells were added to 100 μL of enzyme assay buffer (50 mmol/L potassium phosphate buffer, pH 7.4) containing 0.1 U/mL of horseradish peroxidase and 50 μmol/l Amplex Red. Amplex Red conversion to resorufin (Ex/Em: 570/585 nm) was measured. Maximum fluorescence was reached 4 h after exposure. Pegylated catalase (0.1 U/mL; PEG-CAT) was utilized as a negative control.

### 2.6. Protein Analysis

Notable inflammatory markers were measured using a custom mouse antibody array (Abcam, Waltham, MA, USA). Insulin signaling markers were measured by a rodent phosphorylation array (PEL-AKT-S473-T; PEL-GSK3b-s9-T; Raybiotech, MA, USA). The total protein in the lysates was measured using a BCA Protein Assay Kit (Thermo Fisher Scientific), and samples were added to membranes containing specific capture antibodies and incubated for two hours at room temperature before being incubated again with a second antibody array membrane. Next, biotin-conjugated antibodies were pipetted into each well and incubated. Lastly, streptavidin-HRP, a fluorescent label, was added to each membrane for a final incubation to detect molecule expression. The membranes were imaged using the Odyssey DLx Near-Infrared Fluorescence Imaging System (LI-COR) and quantified using Image J (U.S. National Institutes of Health, Bethesda, MD, USA).

### 2.7. Statistical Methods

Data are presented as means ± SEM. Data were compared with one-way ANOVA and Student’s t-test (Graphpad Prism, Boston, MA, USA). Significance was set at *p* < 0.05.

## 3. Results

### 3.1. Beta-Hydroxybutyrate Mitigates Uric Acid-Induced Changes in Inflammatory Activation and Cell Viability

**Figure 1:**
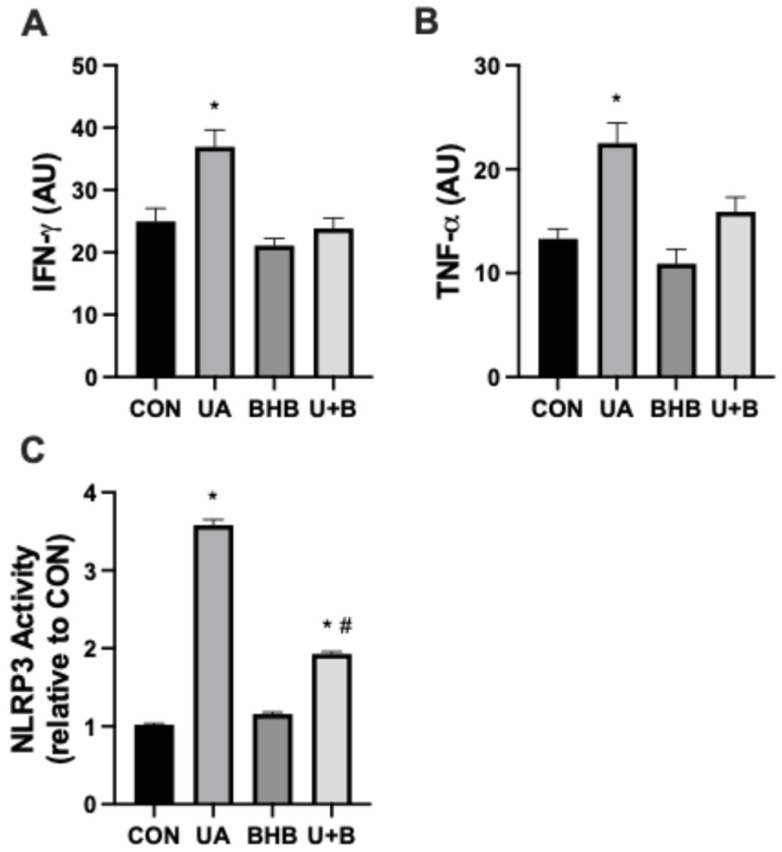
Myoblasts were incubated for 24 h in either uric acid (500 µM), β-hydroxybutyrate (BHB; 5 mM), or both U + B (*n* = 10). Interferon-γ (IFN-γ; (**A**) and tumor necrosis factor-α (TNF-α; (**B**) were measured via dot blot. (**C**) NLRP3 activity was determined via activity assay. * *p* < 0.05 vs. CON. # *p* < 0.05 vs. UA.

Consistent with the established pro-inflammatory effects of uric acid, our results show a marked increase in IFN-γ and TNF-α following uric acid treatment, underscoring the potent inflammatory response elicited by elevated uric acid levels (Figure 1A,B). Interestingly, while BHB alone had no measurable impact on these cytokines, its co-administration with uric acid significantly suppressed both IFN-γ and TNF-α expression. This finding suggests that BHB may selectively counteract uric acid-induced inflammation, potentially by inhibiting pathways specific to the inflammatory cascade initiated by uric acid.

To further investigate the mechanisms underlying this anti-inflammatory effect, we measured NLRP3 inflammasome activity, a key mediator of uric acid-induced inflammation (Figure 1C). As expected, uric acid treatment led to a significant increase in NLRP3 activation relative to control levels, consistent with its role as a potent inflammasome activator. BHB alone had minimal effect on NLRP3 activity; however, co-treatment with BHB and uric acid markedly attenuated NLRP3 activation, reducing it to nearly half of the levels observed with uric acid treatment alone. These results suggest that BHB may exert its anti-inflammatory effects, at least in part, by directly targeting NLRP3 inflammasome activation.

**Figure 2:**
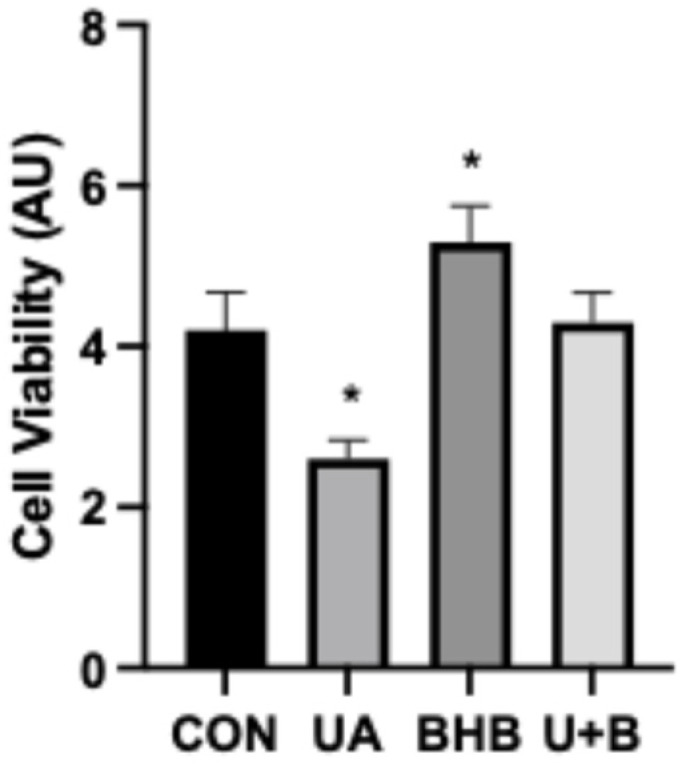
Myoblasts were incubated for 24 h in either uric acid (500 µM), β-hydroxybutyrate (BHB; 5 mM), or both U + B. Cell viability was measured via the MTT assay (*n* = 8). * *p* < 0.05 vs. CON.

As shown in Figure 2, uric acid exposure led to a substantial decrease in cell viability, likely due to the elevated inflammatory milieu. The reduction in viability aligns with prior studies linking inflammatory cytokines to cellular apoptosis and necrosis. Remarkably, BHB co-treatment not only mitigated the adverse effects of uric acid on cell viability but also appeared to enhance cell survival beyond control levels. This protective effect highlights BHB’s potential to maintain cellular integrity in pro-inflammatory environments, possibly by attenuating the cytotoxic impacts of cytokines like TNF-α.

### 3.2. Uric Acid and Ketones Have Differential Effects on Mitochondrial Bioenergetics

**Figure 3:**
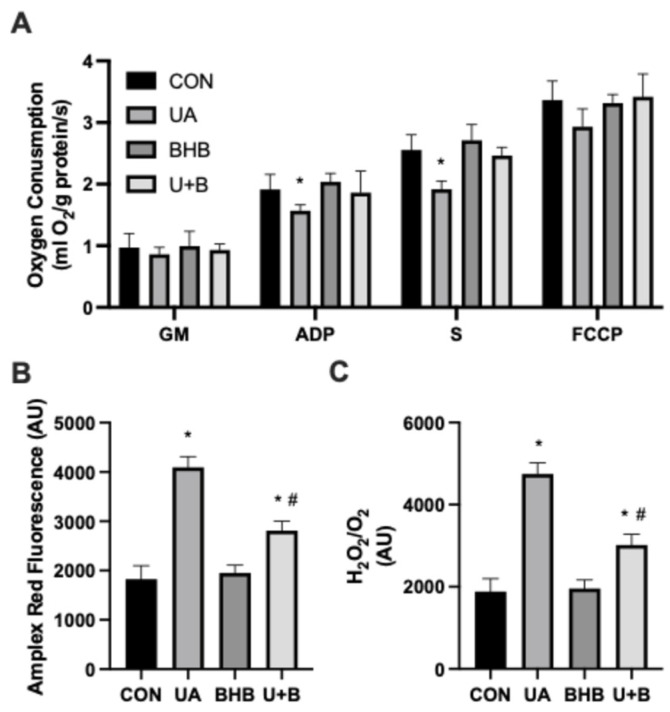
Myoblasts were incubated for 24 h in either uric acid (500 µM), β-hydroxybutyrate (BHB; 5 mM), or both U + B (*n* = 5). Mitochondrial respiration (**A**) was measured by the following strategy: GM indicates glutamate (10 mM) + malate (2 mM); +ADP (2.5 mM); +succinate (S; 10 mM); +FCCP (0.05 μM). H_2_O_2_ generation (**B**) was measured via Amplex Red. The ratio of H_2_O_2_ generation to O_2_ consumed (**C**) was also determined. * *p* < 0.05 for treatment versus CON. # *p* < 0.05 vs. UA.

Uric acid is known to induce pathological changes in mitochondrial function [12]. We confirm that uric acid treatment (UA) is sufficient to significantly reduce mitochondrial respiration in various states (Figure 3A), particularly respiration supported by complex-I-mediated oxidative phosphorylation (“ADP”), as well as complex-II-mediated respiration (“S”), when compared with control cells (CON). Notably, this effect was completely prevented by co-treatment with BHB (U + B). Furthermore, we found that UA elicited a robust increase in H_2_O_2_ generation (as measured via Amplex Red; Figure 3B). While BHB alone elicited no response, the combination (U + B) resulted in a general inhibition, though not to the same levels as CON. Finally, when comparing the generation of H_2_O_2_ with respiration (in basal GM state), this trend continued (Figure 3C).

### 3.3. β-Hydroxybutyrate Protects Insulin Signaling

**Figure 4:**
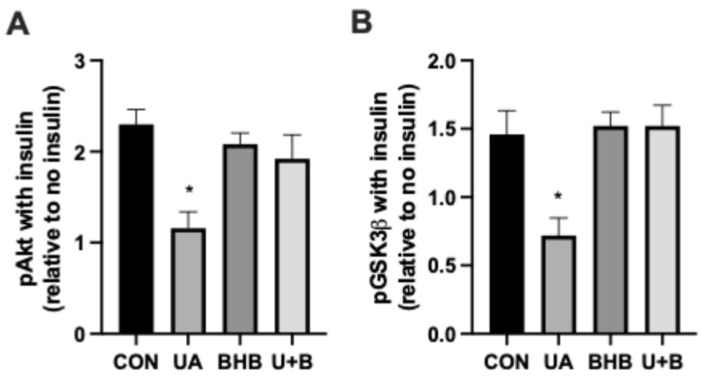
Myoblasts were incubated for 24 h in either uric acid (500 µM), β-hydroxybutyrate (BHB; 5 mM), or both (U + B) followed by 10 min of insulin treatment (100 nM) (*n* = 5). Levels of phosphorylated Akt (pAkt; (**A**) and glycogen synthase kinase 3β (pGSK3β; (**B**) were measured compared with no insulin stimulation. * *p* < 0.05 for treatment vs. CON.

One of the many pathologies that results from increased uric acid in humans is insulin resistance [13]. The mechanism of action for this effect appears to be heavily mediated by the activation of inflammatory pathways, which are known to disrupt insulin signaling [14]. To determine whether the reduced inflammation elicited by co-incubation of cells with UA and BHB (U + B) was sufficient to affect insulin signaling, we determined two primary intermediates of the insulin signaling cascade (pAkt and pGSK3β) following insulin stimulation of cells (Figure 4). We observed that UA treatment resulted in a significant reduction in pAkt (Figure 4A) and pGKS3β (Figure 4B), strong evidence of insulin resistance [15,16]. However, similar to that seen with inflammatory markers, the combination of UA and BHB prevented the decayed signal, resulting in comparable levels of the two phospho-proteins seen in CON cells.

## 4. Discussion

The results of this study provide novel insights into the differential roles of uric acid (UA) and beta-hydroxybutyrate (BHB) in modulating inflammatory pathways, mitochondrial function, and insulin signaling. While the pro-inflammatory and metabolic disturbances caused by elevated uric acid levels have been well documented, our findings underscore the therapeutic potential of BHB in mitigating these detrimental effects, particularly in the context of fundamental features of modern chronic diseases—namely, inflammation, oxidative stress, and insulin resistance.

As expected, we observed that exposure to uric acid significantly upregulated the production of pro-inflammatory cytokines IFN-γ and TNF-α (Figure 1A,B), consistent with previous reports highlighting the inflammatory effects of uric acid and its role in activating the NLRP3 inflammasome [2,3,17]. Notably, BHB alone did not affect cytokine production; however, its co-incubation with UA significantly suppressed these inflammatory markers. This supports the growing body of evidence suggesting that BHB can act as a potent anti-inflammatory agent by directly inhibiting NLRP3 inflammasome activation [3].

To confirm the role of NLRP3 inflammasome activation in UA-induced inflammation, we directly measured NLRP3 activity and found it to be significantly elevated in response to UA treatment (Figure 1C). This aligns with prior studies demonstrating the ability of UA to trigger inflammasome activation, which subsequently drives cytokine production and other inflammatory responses [18]. Importantly, co-treatment with BHB effectively attenuated NLRP3 activation, reducing activity to levels significantly lower than those observed with UA alone. These findings suggest that BHB suppresses the inflammatory cascade by directly targeting inflammasome pathways, consistent with its known inhibitory effects on potassium efflux and mitochondrial ROS—key activators of the NLRP3 inflammasome [3].

Moreover, the reduction in cell viability induced by UA (Figure 2) likely reflects the heightened inflammatory environment, which is known to promote cell death through pathways such as apoptosis and necrosis [19]. Importantly, the co-incubation with BHB not only restored cell viability but even improved it beyond the control levels, suggesting that BHB has a protective effect that extends beyond its anti-inflammatory properties, something we have shown previously in pancreatic β-cells [20]. The fact that BHB not only restores but enhances cell viability beyond control levels suggests it may influence cellular survival pathways. Studies have shown that BHB can activate anti-apoptotic proteins, such as Bcl-2, and inhibit pro-apoptotic factors like Bax in other cell types [21]. This modulation of apoptotic pathways may provide added protection against uric acid-induced cellular stress, supporting BHB’s role as a cytoprotective agent beyond its effects on inflammation and oxidative stress.

Our results further highlight the detrimental impact of UA on mitochondrial bioenergetics, as evidenced by the significant reduction in mitochondrial respiration across several states, particularly in complex I- and complex II-mediated respiration (Figure 3A). This finding is consistent with previous studies demonstrating that UA induces mitochondrial dysfunction, leading to reduced ATP production and increased oxidative stress [22,23]. However, protection of mitochondrial respiration by BHB suggests that ketone bodies may play a critical role in preserving mitochondrial function, potentially by stabilizing mitochondrial membranes and preventing reactive oxygen species (ROS) generation.

In line with this, we observed a marked increase in H_2_O_2_ production following UA treatment (Figure 3B), further supporting the role of oxidative stress in uric acid-induced mitochondrial dysfunction. While BHB alone did not affect H_2_O_2_ levels, its co-incubation with UA significantly reduced oxidative stress, albeit not to control levels. This partial inhibition could be attributed to BHB’s ability to modulate ROS production and maintain mitochondrial integrity, as previously described in studies on ketones and various mitochondria-related outcomes, including oxidative stress [24].

One of the most significant findings of this study is the impact of UA and BHB on insulin signaling. Consistent with prior research, UA significantly impaired insulin signaling, as evidenced by reduced levels of phosphorylated Akt (pAkt) and GSK3beta (pGSK3beta) (Figure 4), both key intermediates in the insulin signaling cascade [22]. There is substantial support for this finding, including in clinical settings. Uric acid inhibitors, used to treat gout and related symptoms, are widely known to improve insulin sensitivity [25]. The addition of BHB to the UA treatment group restored insulin signaling to levels comparable to the control, indicating that the anti-inflammatory effects of BHB extend to the preservation of insulin sensitivity. These findings are in agreement with studies showing that BHB can protect against insulin resistance by reducing inflammatory markers and preserving insulin receptor function [26]. Altogether, including our work, this suggests that UA-induced insulin resistance is heavily mediated through inflammatory pathways that disrupt insulin signaling, corroborating previous findings on the link between inflammation and insulin resistance [27,28,29]. The restoration of insulin signaling by BHB in the presence of uric acid has broader implications for metabolic health. Given that insulin resistance is a hallmark of type 2 diabetes and is closely linked to chronic inflammation, BHB’s ability to protect insulin signaling function could be pivotal for individuals with hyperuricemia and concurrent insulin resistance. This dual action on both inflammation and insulin signaling positions BHB as a promising candidate for metabolic interventions aimed at reducing the risk of type 2 diabetes and improving metabolic flexibility.

While our findings provide meaningful insights into the interplay between uric acid, BHB, and key inflammatory, metabolic, and mitochondrial events, there are important limitations to consider. First, this study was conducted in a cell culture model, which, while valuable for isolating specific mechanisms, lacks the complexity of whole-organism systems where factors such as hormonal regulation, immune system interactions, and tissue-specific responses play critical roles. The extrapolation of our results to in vivo contexts therefore requires caution. Additionally, the concentrations of uric acid and BHB used in this study, while reflective of pathological and therapeutic ranges, may not fully capture the dynamic fluctuations seen in vivo. Finally, while BHB’s protective effects are evident, the precise mechanisms underlying its influence on cell viability, mitochondrial function, and insulin signaling remain incompletely understood and warrant further investigation using more advanced models, including animal studies and clinical trials. Future research should aim to address these gaps to better translate these findings into therapeutic strategies.

## 5. Conclusions

In summary, our study demonstrates that uric acid induces significant inflammatory and metabolic disturbances, including increased pro-inflammatory cytokine production, mitochondrial dysfunction, oxidative stress, and insulin resistance. However, BHB effectively mitigates these detrimental effects, providing evidence for its therapeutic potential in conditions characterized by hyperuricemia and metabolic disturbances. Further research is warranted to explore the clinical implications of BHB in the treatment of metabolic syndrome, type 2 diabetes, and cardiovascular diseases.

## Data Availability

Data can be made available upon request.
